# MesenchymAl stromal cells for Traumatic bRain Injury (MATRIx): a study protocol for a multicenter, double-blind, randomised, placebo-controlled phase II trial

**DOI:** 10.1186/s40635-023-00535-1

**Published:** 2023-08-25

**Authors:** Elisa R. Zanier, Francesca Pischiutta, Eliana Rulli, Alessia Vargiolu, Francesca Elli, Paolo Gritti, Giuseppe Gaipa, Daniela Belotti, Gianpaolo Basso, Tommaso Zoerle, Nino Stocchetti, Giuseppe Citerio, on behalf of the MATRIx Study group

**Affiliations:** 1 Department of Acute Brain and Cardiovascular Injury, Istituto di Ricerche Farmacologiche Mario Negri IRCCS, Milan, Italy; 2 Department of Clinical Oncology, Istituto di Ricerche Farmacologiche Mario Negri IRCCS, Milan, Italy; 3 School of Medicine and Surgery, University of Milano-Bicocca, Milan, Italy; 4 Neurological Intensive Care Unit, Department of Neurosciences, Fondazione IRCCS San Gerardo dei Tintori, Monza, Italy; 5 Department of Anesthesia, Emergency and Critical Care Medicine, ASST Ospedale Papa Giovanni XXIII, Bergamo, Italy; 6 M. Tettamanti Research Center, Fondazione IRCCS San Gerardo dei Tintori, Monza, Italy; 7 Department of Neurosciences, Neuroradiology, Fondazione IRCCS San Gerardo dei Tintori, Monza, Italy; 8 Neuroscience Intensive Care Unit, Department of Anaesthesia and Critical Care, Fondazione IRCCS Ca’ Granda-Ospedale Maggiore Policlinico, Milan, Italy; 9 Department of Pathophysiology and Transplants, University of Milan, Milan, Italy

**Keywords:** Traumatic brain injury, Mesenchymal stromal cells, Cell therapy, Neurogenesis and synaptic plasticity, Inflammation

## Abstract

**Background:**

Traumatic brain injury (TBI) is a significant cause of death and disability, with no effective neuroprotective drugs currently available for its treatment. Mesenchymal stromal cell (MSC)-based therapy shows promise as MSCs release various soluble factors that can enhance the injury microenvironment through processes, such as immunomodulation, neuroprotection, and brain repair. Preclinical studies across different TBI models and severities have demonstrated that MSCs can improve functional and structural outcomes. Moreover, clinical evidence supports the safety of third-party donor bank-stored MSCs in adult subjects. Building on this preclinical and clinical data, we present the protocol for an academic, investigator-initiated, multicenter, double-blind, randomised, placebo-controlled, adaptive phase II dose-finding study aiming to evaluate the safety and efficacy of intravenous administration of allogeneic bone marrow-derived MSCs to severe TBI patients within 48 h of injury.

**Methods/design:**

The study will be conducted in two steps. Step 1 will enrol 42 patients, randomised in a 1:1:1 ratio to receive 80 million MSCs, 160 million MSCs or a placebo to establish safety and identify the most promising dose. Step 2 will enrol an additional 36 patients, randomised in a 1:1 ratio to receive the selected dose of MSCs or placebo. The activity of MSCs will be assessed by quantifying the plasmatic levels of neurofilament light (NfL) at 14 days as a biomarker of neuronal damage. It could be a significant breakthrough if the study demonstrates the safety and efficacy of MSC-based therapy for severe TBI patients. The results of this trial could inform the design of a phase III clinical trial aimed at establishing the efficacy of the first neurorestorative therapy for TBI.

**Discussion:**

Overall, the MATRIx trial is a critical step towards developing an effective treatment for TBI, which could significantly improve the lives of millions worldwide affected by this debilitating condition.

*Trial Registration* EudraCT: 2022-000680-49.

## Background and rationale of the study

Traumatic brain injury (TBI) is a leading cause of death and disability, with a high burden on patients, their families, and society [1–4]. Long-term mortality in TBI is substantial, and TBI survivors have a life expectancy shortened by 6 years [5]. TBI results in a progressive polypathology involving interconnected processes, such as neuroinflammation, cortical degeneration, white matter damage, neuronal loss, and blood–brain barrier (BBB) dysfunction [6]. As a consequence, approximately 30% of patients experience the development of progressive neurological deficits [7].

Recently, a large cohort observational study aimed at better characterising TBI across Europe showed that functional outcome is still poor for severe TBI, essentially unchanged in the last 30 years [2]. Treatment of TBI patients has not changed much in the last 20 years, consisting only of supportive therapy directed at prevention, early detection and treatment of second insults [8–10]. Neuroprotective treatments are urgently needed for this condition. It is conceivable that multiple therapeutic targets may need to be addressed simultaneously to interfere with the natural evolution of brain damage after TBI and improve patient’s outcomes. In this contest, mesenchymal stromal cells (MSCs) are ideal candidates [11], since they act on multiple protection and repair mechanisms, improving structural and functional outcomes after experimental TBI.

### MSC pleiotropic effects

MSCs are multipotent progenitor cells first isolated from bone marrow [12], and subsequently from many other sources, including adipose tissue and birth-related tissues [13]. MSCs are attractive candidates for cell therapy because of their ease of isolation and ex vivo expansion, their low immunogenicity and high immunosuppressive activity [14]. MSCs release multiple soluble factors, including growth factors, chemokines, cytokines and exosomes/extracellular vesicles acting in a paracrine fashion, promoting cell survival and reducing neuronal apoptosis [15–17], modulating inflammatory response [18–22], promoting angiogenesis [23–26] neurogenesis and synaptic plasticity [27–29], with a global amelioration of the injury microenvironment and outcome [14, 30, 31].

### Preclinical evidence

 The first experimental study assessing MSC protective effects in TBI was published about 20 years ago [32]. Since then, several experimental studies from our group and others have demonstrated MSC therapeutic efficacy in TBI models. We recently ran a systematic review and meta-analysis to grade preclinical evidence of MSC efficacy in TBI [33]. Our review documents a substantial and favourable effect of MSCs on sensorimotor and cognitive functions and anatomical damage in preclinical models of TBI. In detail, we reported a significant improvement of sensorimotor and cognitive function already at acute (1–2 week post-transplant) and more significant effect size at later (4–5 week post-transplant) timepoints, while the efficacy of MSCs on anatomical damage only became evident 4–5 week post-treatment (Table 1). The efficacy of MSCs on neurological outcomes is robust across many variables, including MSC features (source and type of transplant), treatment protocol (time and route of administration) and experimental models (host species and TBI model). Data indicate a therapeutic window extending over the first week after TBI and a smaller effect size when MSCs are given months after TBI. MSC treatment at chronic stages is more manageable in terms of feasibility but has limited neuroprotective potential. Thus, acute MSC treatment is highly desirable to maximise neuroprotection and promote neurorestorative processes [11]. In addition, preclinical evidence showed that the presence of the cells in the lesion site is not needed to confer neuroprotection [34], with similar efficacy observed after central or systemic MSC administration [24]. Thus, data support a paracrine action by MSC-released soluble factors in remodelling the injury environment. These findings have important clinical implications, since they allow proposing a systemic delivery protocol through the central venous line with direct advantages regarding invasiveness and patient selection. In clinical trials, systemic delivery is the most commonly used administration route, with proven safety in several pathological conditions, including critically ill patients with acute respiratory distress syndrome [35, 36].

**Table 1:** Summary results of meta-analysis data on preclinical evidence of MSC efficacy in TBI models. Data extracted from [34]

	Sensorimotor function	Cognitive function	Anatomical damage
**Acute**			
Number of comparisons	56	20	14
Absolute effect size [95% CI]	1.14 [− 1.41, − 0.88]	0.91 [0.49, 1.33]	0.24 [− 0.04, 0.51]
p value	< 0.0001	< 0.0001	0.092
**Chronic**			
Number of comparisons	29	23	20
Absolute effect size [95% CI]	1.66 [− 2.04, − 1.29]	2.11 [1.56, 2.66]	1.34 [0.89, 1.80]
p value	< 0.0001	< 0.0001	< 0.0001

Studies in TBI rodents indicate that effective doses of MSCs infused intravenously (IV) range from 1 to 4 million cells. Using allometric scaling as recommended by the Food and Drug Administration [37, 38], effective doses in patients could range from 0.8 to 3.2 million MSCs/Kg or 56–224 million MSCs per subject, considering a median patient weight of 70 kg. A recent review discusses challenges with MSC clinical trials, including those related to the large range of tested doses. Focusing on the IV route, authors suggest a minimal effective dose (MED) between 70 and 190 million cells (adult, 70 kg weight, corresponding to 1–2.7 million cells/Kg) and propose an inverted U-shaped dose–response curve, with doses below 70 million likely ineffective and doses above 200 million less effective [39]. Accordingly, the first part of our clinical study will test two doses of either 80 or 160 million MSCs (1.1 and 2.2 million cells/Kg). Notably, doses even higher than these have already been tested and proved safe in the START trial (10 × 106 cells/Kg, corresponding to 700 million cells) [40].

### Neurofilament light as a pharmacodynamic marker

We designed an experimental medicine study, relying on the circulating marker of axonal damage neurofilament light (NfL) to assess MSC efficacy. In the context of TBI, measurement of circulating NfL has shown prognostic utility after mild [41, 42] and severe TBI [43]. Compared to GFAP, UCH-L1, and S100B that show prognostic values (GFAP [42, 44], UCH-L1 [44, 45], S100B [45]) and may inform on computed tomography (CT) abnormalities (as for GFAP [46–48]), NfL has the advantage of different temporal trajectory with a progressive increase in plasma levels over the first 2 weeks after injury [49, 50]. Importantly, initial serum NfL levels are predictive of clinical outcome (Glasgow Outcome Scale extended score, GOSE) at 12 months after injury in severe TBI [49] and correlate with brain volumes and magnetic resonance imaging (MRI) measures of white matter (WM) axonal integrity [50, 51]. NfL has shown validity as a pharmacodynamic biomarker in monitoring treatment response after acute or chronic neurological disorders [52–54]. Thus, NfL is now emerging as a blood biomarker with diagnostic and prognostic utility for TBI patients and may inform the efficacy of experimental medicine studies.

## Study design and objectives

**Fig. 1:**
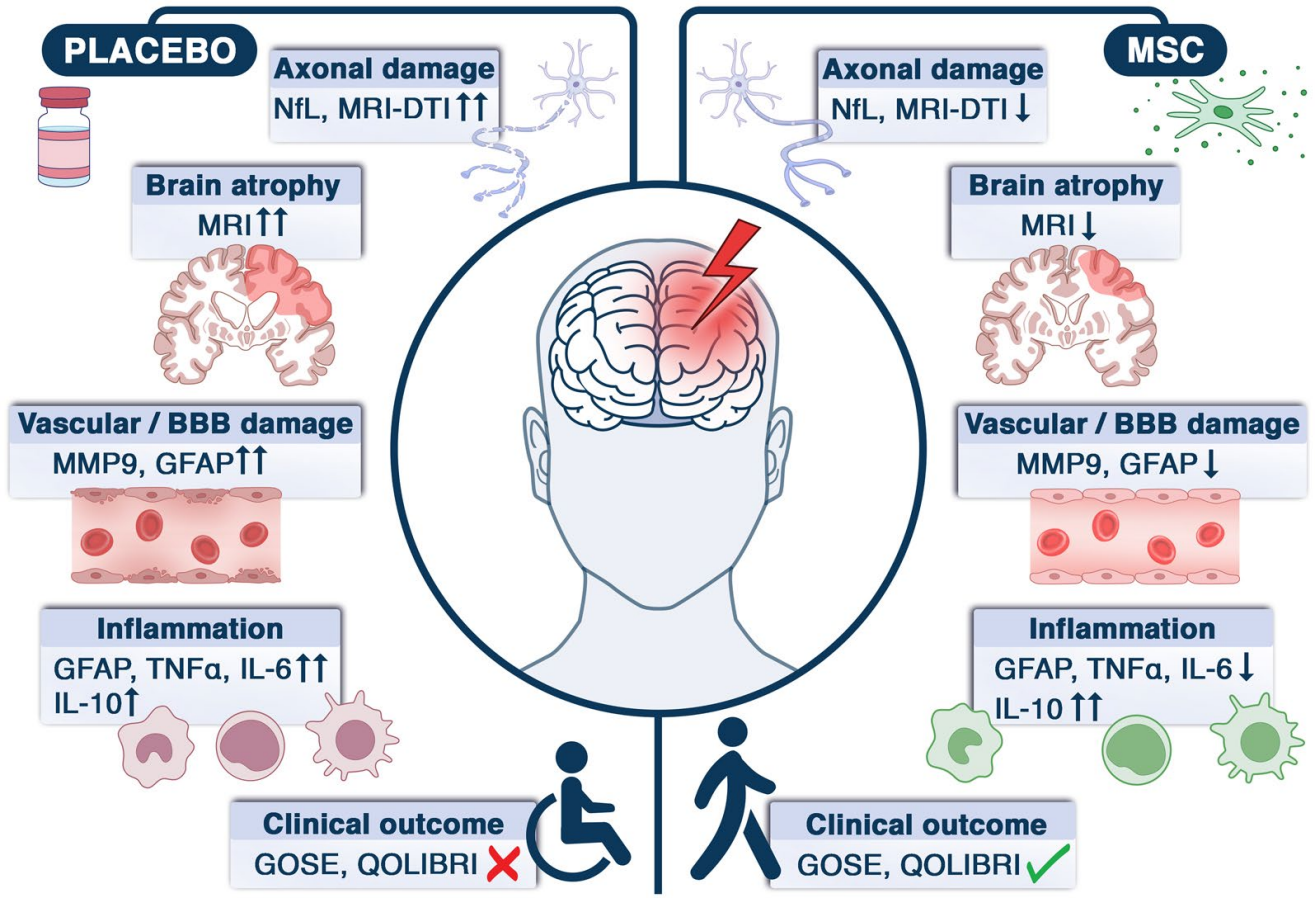
Representation of the expected effects of MSC treatment on primary and secondary outcome measures.

This is a multicenter, double-blind, randomised, placebo-controlled, adaptive phase II dose-finding study to assess the safety and biological effects of the allogeneic bone marrow-derived MSCs, administered IV in severe TBI patients within 48 h from injury. The study will be conducted in two steps. In step 1, patients will be randomised with a 1:1:1 ratio to receive 80 million MSCs, 160 million MSCs, or a placebo to establish safety and select the most promising dose. In step 2, patients will be randomised (1:1) to receive the selected dose of MSCs or placebo to assess MSC activity by quantifying the plasmatic levels of the NfL at 14 days as a biomarker of neuronal damage and by exploring their pleiotropic actions via advanced neuroimaging studies, blood measurements of immune changes and assessment of functional outcome (Fig. 1).

### Primary objectives

The study aims to define:

- If MSCs, administered at a dosage of 80 or 160 million cells, are safe in patients with severe TBI. Safety will be evaluated as the number of patients experiencing at least one serious adverse drug reaction (SADR).
- If MSCs, at the dosage found to be safe and more promising in terms of activity at a planned interim analysis, decrease the plasmatic NfL biomarker of brain damage at 14 days compared to placebo.

### Secondary objectives

Secondary objectives will assess the biological activity of intravenous infusions of allogeneic MSCs in terms of modification of the following clinical variables and biological parameters:

1. Brain injury evolution and white matter damage by advanced longitudinal MRI (with morphological sequences including T1, T2 as well as FLAIR, SWI, DWI, and DTI for quantification of traumatic axonal injury) acutely (4 days) subacutely (14 days) and at 6 month post-TBI to provide a detailed description in atrophy, diffusion and myelin integrity.
2. Brain immunomodulatory changes by temporal profiling (daily for 3 days after TBI, at days 7 and 14 and at 1, 6 and 12 months) of circulating biomarkers of structural damage (including NfL and GFAP), neuroinflammation (i.e., IL-6, IL-10, TNFα) and vascular integrity (i.e., MMP9).
3. Clinical outcome by a structured clinical and neuropsychological outcome assessment at both 6 and 12 months, by Glasgow Outcome Scale Extended (GOSE) and Quality of life after brain injury (QOLIBRI) test.

**Fig. 2:**
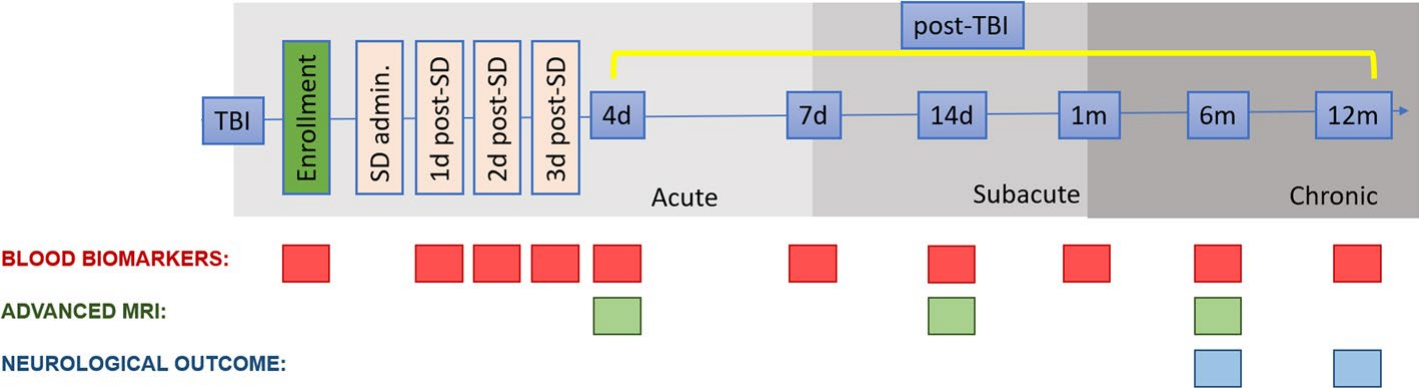
Timepoints for longitudinal assessment of secondary outcomes. *SD*  study drug

The timepoints for the secondary outcome measurements are shown in Fig. 2.

## Methods

### Study setting

About 78 adult TBI patients admitted in intensive care units (ICU) will be enrolled in 3 centers: the Fondazione IRCCS San Gerardo dei Tintori Monza, the Fondazione IRCCS Cà Granda Ospedale Maggiore Policlinico Milano and the ASST Ospedale Papa Giovanni XXIII Bergamo. Patients will be enrolled if fulfilling the eligibility criteria listed in Table 2.

**Table 2:** Eligibility criteria

Inclusion criteria	Exclusion criteria
– Age: 18–70 years (inclusive) – Clinical frailty index < 5 [60] – Evidence of TBI confirmed by abnormalities consistent with trauma on CT scan upon admission (Marshall’s CT Classification > 1) – Study drug (MSCs/placebo) administration start within 48 h from TBI – GCS ≤ 8 at recruitment and at least one pupil reactive to light – ICP monitoring, already inserted or planned for clinical indications – Weight < 100 kg and > 40 kg	– Motor GCS > 5 at recruitment – High likelihood (> 85%) of death in the first 48 h calculated by IMPACT calculator [61] on early admission data – Bilateral unreactive mydriasis – Opening ICP > 40 mmHg – Known history of prior brain injury, psychiatric disorder, neurological impairment and/or deficit – Brain penetrating injury – Spinal cord injury – Epilepsy requiring ongoing anti-convulsant therapy – Severe organ failure (including PaO_2_/FiO_2_ < 200 and shock) – Recent serious infectious process requiring ICU admission – Cancer (ongoing) – Immunosuppression – Human immunodeficiency virus – Positive urine pregnancy test or breastfeeding – Known risk/history of coagulopathy and thromboembolism – Pre-existing and severe: ▪ Lung disease (such as asthma, chronic obstructive pulmonary disease) ▪ Heart dysfunction (as heart failure and reduced cardiac output) ▪ Liver insufficiency (as cirrhosis) ▪ Kidney insufficiency ▪ and other organ severe abnormalities – Known hypersensitivity to excipients used in the formulation (dimethyl sulfoxide, DMSO; citrate-dextrose solution, ACD) – Participation in a concurrent interventional study

CT, computed tomography; GCS, Glasgow Coma Scale; ICP, intracranial pressure; ICU, intensive care unit; IMPACT, international mission for prognosis and clinical trial; MSCs, mesenchymal stromal cells; TBI, traumatic brain injury

MSCs will be provided by the cell factory Stefano Verri (Fondazione IRCCS San Gerardo dei Tintori Monza) authorised by Agenzia Italiana del Farmaco (AIFA) for the manufacturing and quality control of cell-based medicinal products for advanced therapies.

Longitudinal analysis of the effects of MSCs on brain structural and neuroimmunomodulatory changes by blood biomarker in relation to brain-advanced neuroimaging trajectories and clinical outcomes will be performed at Istituto di Ricerche Farmacologiche Mario Negri IRCCS, Milano.

### Informed consent procedures

Consent will be obtained according to the Declaration of Helsinki guidelines and local regulations from each subject participating in this study and/or his/her legally designated representative/proxy.

Since comatose patients could not provide informed consent at the time of study recruitment, each center referred to the local/national law on the lack of capacity. The requirements established by the Standards of Good Clinical Practice (GCP) prepared by the International Conference on Harmonization of Technical Requirements for the registration of pharmaceutical products for human use in relation to consent in emergency situations (Decree of the Ministry of Health July 15 1997) will be applied.

The investigator/delegate will share with the family members the purpose and risks/benefits of the study, the possible study-related adverse events, the study-specific procedures, the duration of the study, and the schedule of planned follow-up visits.

If the patient will regain consciousness and the ability to provide consent, he/she will be required either to give informed consent to use of acute and follow-up data or to refuse to participate in the research. The patient will be completely free to withdraw from the study at any time for any reason without having to provide any justification.

### Study drug production and preparation

Allogeneic MSCs will be prepared according to Good Manufacturing Procedures (GMP) guidelines at Laboratorio Stefano Verri [55], Fondazione IRCCS San Gerardo dei Tintori, Monza. MSCs are stored in vials containing 40 million MSCs each in 4.5 ml of a storage solution (2.5 ml albumin 20%, 1.1 ml normal saline solution, 0.45 ml citrate-dextrose solution (ACD), 0.45 ml dimethyl sulfoxide (DMSO)). Vials containing only 4.5 ml of storage solution will be prepared and stored similarly. Vials are stored in the vapour phase of a liquid nitrogen tank until the time of administration or sent to dislocated sites via a GMP-qualified local supplier, ensuring the product's safety and quality during transportation.

After patient enrolment, the study drug will be prepared for administration. A total of 4 vials/patients (combined to obtain placebo, MSCs 80 million, MSCs 160 million, in a total of 18 ml) will be thawed under a class A laminar hood near the ICU and diluted 1:2 in saline solution for a total of 36 ml. Treatments will be collected in a syringe, connected to the patient's central venous line and administered within 15 min from the preparation. The administration of the treatment will be masked.

### Management of adverse events

An adverse event (AE) is any untoward medical occurrence in a clinical trial participant administered a medicinal product and which does not necessarily have a causal relationship with the study treatment. A serious adverse event (SAE) is an AE that fulfils one or more of the following criteria:

- Results in death
- Is immediately life-threatening
- Requires in-patient hospitalisation or prolongation of existing hospitalisation
- Results in persistent or significant disability or incapacity
- Is a congenital abnormality or birth defect in offspring of the patient
- Is an important medical event that may jeopardise the patient or require medical intervention to prevent one of the above outcomes.

A SADR is an SAE related to the study drug.

The site investigator is responsible for assessing the severity, seriousness and relationship between each AE and the study drug, as soon as AEs occur.

### Statistical design and sample size

The statistical study design is conceived in 2 steps.

#### Step 1

An interim safety analysis will be performed to evaluate separately in each of the two experimental dosage groups, at a one-sided type I error rate of 10%, whether more than 30% of patients experience at least one SADR within 14 days from treatment and at the same time at a power of 80% whether 5% or less of the patients do not experience any SADR. Adopting the Fleming design with A’Hern’s approach, 12 evaluable patients will be analysed in each group. The maximum number of patients experiencing at least one SADR to observe in each experimental group is 1 out of 12, since this result is associated with an upper limit of the 80% exact confidence interval of 28.8%. The experimental treatment would be considered safe for this step only if none or one patient experimented with a SADR.

According to the above roles:

- If both experimental treatments are unsafe, the study will be stopped for safety issues, and step 2 will not be performed.
- If one experimental treatment is considered safe and the other not, only the safe arm will proceed with step 2 of the study.
- If both the experimental dosages are safe, the more active schedule will be selected for step 2.

The more active schedule will be defined in terms of the proportion of patients who reaches an NfL increase at 14 days equal to or lower than the baseline level by five folds (defined as “responder patient”). This cutoff has been determined based on longitudinal quantification provided by BIO-AX-TBI collaborators [50], showing a median increase of 12.8-fold at 14 days compared to baseline, with the first quartile equal to a fivefold increase. The experimental arm with a higher frequency of responders will be selected as the most promising in terms of activity and it will be chosen for the second step. In the case of an equal number of responder patients in the two experimental arms, the cutoff defining the response will be increased by 1 unit iteratively, since a difference between the two experimental arms will be observed. This assessment will only be performed on the MSC-treated arms; the control group will not be considered. According to Simon, Wittes and Ellemberg randomised phase II design [56], the study has more than an 80% probability of correctly selecting the more active schedule when the proportion of responder patients is 15% higher in the first of the two treatment schedules.

#### Step 2

The endpoint will be the NfL at 14 days. The final analysis of biological activity will be performed by increasing the sample size to 27 evaluable patients in the experimental and control arms. The study is designed to detect an effect size of 0.59 on the logarithmic scale of the experimental to control arm, setting a one-sided type I error rate of 10% and a power of 80%.

Therefore, the total number of evaluable patients to be analysed will be 66 (27 in the control arm, 12 in the experimental arm stopping at the first step and 27 in the experimental arm reaching the second step). Expecting 13% of deaths of TBI patients in ICU [2], the number of patients to be randomised is about 78 (32 in the control arm, 14 in the experimental arm stopping at the first step and 32 in the experimental arm reaching the second step). After the trial closure after the first step, the number of patients to randomise will be about 42 (14 in each arm).

### Random allocation procedure

Patients will be randomly assigned to one of the three treatment groups with an equal probability of assignment to each treatment (allocation ratio 1:1:1 for the first step and 1:1 for the second step). The study biostatistician will prepare the sequence of treatments according to a randomised permuted blocks procedure stratified by experimental centers. The randomisation list will be generated using SAS 9.4 release. The randomised allocation of treatment will be centralised and managed by the cell factory responsible to label the treatment according to the randomisation list.

**Fig. 3:**
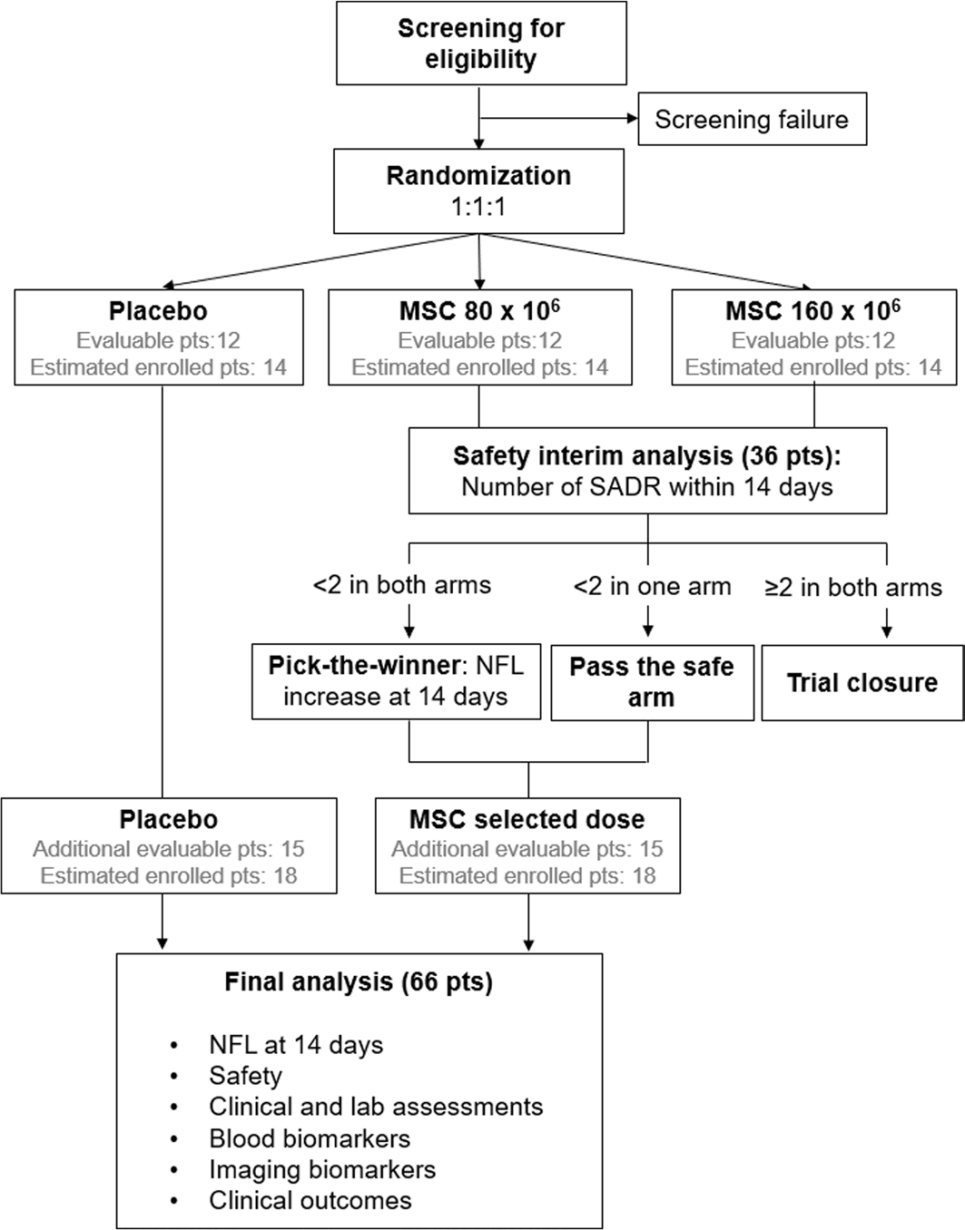
Flowchart of the MATRIx trial

A schematic representation of the trial is depicted in Fig. 3 (flow chart).

### Blinding

Patients and investigators are blinded to the treatment. Treatment assignment will be known only to the Sponsor Data Management Team, the Cell Factory and the study's biostatistician. The MSC and the placebo will have identical appearance and consistency. The Investigators will receive sealed emergency envelopes allocated to their site. Each envelope is labelled with the protocol acronym and a treatment identification code. Inside the envelope, the respective treatment arm is recorded. The Investigator is authorised to unseal the envelope only in case of a medical emergency when adequate treatment of the concerned patient requires immediate knowledge of the trial treatment. If it felt useful, the investigator may first discuss options with the other investigators and the scientific coordinator from Sponsor before unblinding the subject’s treatment assignment. The study pharmacovigilance dedicated staff retains the right to break the code for SAEs that are unexpected and suspected to be causally related to the investigational product and potentially require expedited reporting to regulatory authorities. The subject which has been unblinded may continue study treatment according to clinical judgement.

### Data analysis

The analysis of primary activity outcomes will be performed on the per-protocol (PP) population, including all randomised patients, without major eligibility violations criteria, who received the allocated treatment as specified in the protocol and with a follow-up of at least 14 days. Major violations in the eligibility criteria will be evaluated on a case-by-case basis in a pre-analysis meeting to define the population.

The safety analysis population will include all patients who have received the treatment. Each patient will be analysed in the arm received.

Frequency and percentages will describe the proportion of patients in each experimental group experiencing at least one SADR within 14 days from treatment administration.

In terms of NfL change from baseline to 14 days, the proportion of responder patients will be described through frequency and percentages. Regarding the NfL measured at 14 days, a one-sided Student's *t* test on the logarithmic scale will be performed to compare the experimental and control arms. Assumptions of Student's *t* test (i.e., normality of residuals and homoscedasticity) will be checked on a log scale using graphical (i.e., histograms and normal quantile plots) and formal methods (Shapiro–Wilk test for normality and Levene's test for equal variance). In case of failed assumptions, a proper data transformation will be considered. Univariable and multivariable generalised linear regression models will be used to evaluate the impact of the potential prognostic factors on the NfL at 14 days. Univariable and multivariable generalised linear mixed regression models for repeated measures will be used to evaluate the impact of treatment on longitudinal data.

Loss to follow-up patients and patients with missing values will contribute to the analysis of the secondary outcomes only for the time data are available. Univariable and multivariable generalised linear mixed regression models for repeated measures will be used to evaluate the impact of treatment on longitudinal MRI and blood biomarkers, and functional outcome.

Safety assessment will be mainly based on adverse reactions (ARs) and the frequency and nature of the serious adverse events (SAEs) and will be conducted on the safety analysis set. For each patient and for each type of adverse event, the worst degree ever suffered will be used for the analysis. SAEs will be summarised by presenting the number and percentage of patients having any SAE and having an SAE in each system organ class. Other information collected (e.g., severity or suspected relationship to study medication) will be listed as appropriate.

## Discussion

There are no effective neuroprotective treatments for TBI. Decades of research in TBI experimental models provide evidence of MSC efficacy across multiple cell sources, and routes of administration with a large therapeutic window. MSCs have be shown not to be immunogenic allowing the design of an allogenic transplant in immunocompetent patients. Allogeneic MSCs infused at chronic stages in TBI patients show preliminary evidence of clinical benefits [57, 58]. Here, we propose to target the acute detrimental TBI phase using allogeneic MSCs. During the coronavirus disease 2019 pandemic, several studies have confirmed the safety of MSC treatment infused in critically ill patients [59], allowing the design of a phase II randomised placebo controlled clinical trial in acute severe TBI patients. The MATRIx trial will define the safety of MSC treatment in acute severe TBI patients and provide initial evidence of MSC biological activity in blunting NfL in plasma as the intermediate biochemical endpoint of axonal damage. We will explore the MSC pleiotropic effects by investigating the temporal profiling of circulating biomarkers of structural damage (including NfL and GFAP), neuroinflammation (i.e., IL-6, IL-10, TNFα) and vascular integrity (i.e., MMP9). Furthermore, a detailed longitudinal description of brain-advanced neuroimaging (MRI) trajectories will document MSC ability to affect the anatomical damage and white matter integrity. At last, evaluation of clinical outcomes at chronic stages will be indicative of MSC impact on patient quality of life.

Success in this project will pave the way for a subsequent phase III trial to test MSC efficacy on neurological development in severe TBI.

## Data Availability

The integral version of the protocol will be available to investigators upon reasonable request.
